# Natural killer cells strengthen antitumor activity of cisplatin by immunomodulation and ameliorate cisplatin-induced side effects

**DOI:** 10.1007/s11255-023-03650-w

**Published:** 2023-05-30

**Authors:** Zhu Wang, Zhan Yang, Changbao Qu, Jinmin Li, Xiaolu Wang

**Affiliations:** 1 Department of Urology, The Second Hospital of Hebei Medical University, Shijiazhuang, 050000 China; 2 Department of Pediatric Surgery, The Second Hospital of Hebei Medical University, Shijiazhuang, 050000 China

**Keywords:** Natural killer cell, Cisplatin, Bladder cancer, Immunomodulation, Treatment

## Abstract

**Purpose:**

Cisplatin-based chemotherapy is now an important treatment for improving bladder cancer prognosis. However, challenges in clinical treatment remain due to the numerous side effects of chemotherapy. Natural killer (NK) cells regulate certain immune responses and play a significant role in tumor surveillance and control. The efficacy of NK cells combined with cisplatin for chemoimmunotherapy in bladder cancer remains poorly understood.

**Methods:**

In this study, we established an MB49 tumor-bearing mouse model, tumor growth was measured in a control group and in groups treated with cisplatin, NK cells or both. Organ indices, biochemical indicators of blood serum, and expression of apoptotic proteins were used to assess the extent of organ damage. ELISA and immunohistochemistry were used to analyze the levels of immune cells and cytokine expression in serum, spleen, and tumor tissue.

**Results:**

NK cells combined with cisplatin exhibited better antitumor activity. NK cells also alleviated the organ damage caused by cisplatin and improved the survival rate. Treatment with NK cells increased the expression of IL-2 and IFN-γ as well as the number of CD4 + T cells. Additionally, cisplatin increased the expression of natural killer group 2, member D (NKG2D) ligands thus activating NK cells to kill tumor cells.

**Conclusion:**

NK cells could alleviate the side effects of cisplatin treatment and enhance antitumor activity. The combination of NK cells and cisplatin thus provides a promising option for chemoimmunotherapy for bladder cancer.

**Supplementary Information:**

The online version contains supplementary material available at 10.1007/s11255-023-03650-w.

## Introduction

Cancer substantially impairs human health and endangers life. Bladder cancer is one of the most common urological cancers, with approximately 550,000 new cases diagnosed globally each year [1, 2]. In recent decades, the overall incidence of bladder cancer has increased owing to the potential effects of carcinogens released by industry, population aging, and factors such as smoking [3, 4]. Bladder cancer is the 9th most common cancer overall, the 7th most common malignant tumor in men (9.5/100,000), and after the 10th most common malignant tumor in women (2.410/100,000). In terms of the mortality rate, bladder cancer ranks 13th among malignancies, at 3.2/100,000 in men and 0.9/100,000 in women. The most common early symptoms of bladder cancer are painless gross hematuria, urinary urgency or frequency, and dysuria. The disease has a long course and a poor prognosis, which seriously affects patients’ quality of life [5]. Surgery is currently the primary treatment for bladder cancer, but it has a high postoperative recurrence rate of approximately two-thirds [6]. About 25% of patients exhibit muscle invasion and distant metastasis at the time of initial diagnosis. Chemotherapy is regarded as one of the most efficient methods for treating bladder cancer [7]. Cisplatin is a platinum-containing anticancer drug that was discovered in the early 1960s and is used to treat various solid tumors [8–10]. The first-line treatment for bladder cancer is cisplatin-based chemotherapy. Although chemotherapy has significantly improved the prognosis of patients, challenges in clinical treatment remain because of the numerous side effects of chemotherapy drugs, including immunosuppression, organ toxicity, and drug resistance. There is thus an urgent need to develop adjuvant antitumor agents with better therapeutic efficacy and less toxicity in order to improve patients’ quality of life.

Natural killer (NK) cells are a crucial element of the body’s natural immune system, and they play an important role in fighting against illnesses [11, 12]. NK cells have immune clearance and immune surveillance functions, and they can destroy target cells in the absence of an antigen stimulus. Recent research has shown that NK cells have immunotherapeutic potential in many cancer types, thereby being crucial for tumor surveillance and control, effectively killing tumors, and producing cytokines and chemokines that regulate certain immune responses [13]. This has gradually revealed the potential of NK cells as a target for cancer immunotherapy. Several cancer types, such as bladder cancer, clear cell renal cell carcinoma, invasive breast cancer, colon adenocarcinoma, endometrial carcinoma of the uterine corpus, and thyroid cancer, are related to NK cells deficiency, resulting in poor clinical outcomes [14]. Moreover, an imbalance of immunomodulatory signaling in NK cells has been observed in many cancers [13, 15, 16], while some malignancies can evade immune monitoring due to NK cells malfunction [17–19]. The cytotoxic function and phenotype of NK cells are inextricably linked, and the balance of inhibitory and activating signals determines how these cells function as effectors [20]. Therefore, restoring NK cells function is one potential immunotherapeutic strategy for treating patients with cancer [21–23].

At present, the potential of NK cells as a therapeutic target provides hope for cancer patients as they are suffering from the side effects of chemotherapy and drug resistance. According to a previous report, the combination of NK cells and gemcitabine was effective for treating hepatocellular carcinoma [24]. However, few studies have been reported on the application of NK cells in combination with cisplatin for chemoimmunotherapy in bladder cancer. In this research, we sought to employ an MB49 tumor-bearing mouse model and NK cells to illustrate the antitumor efficacy, immunoregulatory potential, and potential mechanisms of cisplatin combined with NK cells against bladder cancer.

## Materials and methods

### Animal studies

Six- to seven-week-old male C57BL/6N mice weighing 18–22 g were purchased from Vital River Laboratory Animal Technology Co., Ltd. (Beijing, China). The mice were housed in identical conditions at the Laboratory Animal Center of the Second Hospital of Hebei Medical University. A total of 3 × 10^6^ MB49 cells were subcutaneously injected into the right flank of these mice. MB49 cells were resuspended in 100 μl of DMEM without serum. When the tumor volume reached approximately 50 mm^3^, the mice were randomized into four groups (n = 15), and treatment was initiated when the mean tumor volume reached 100–200 mm^3^. The first group was a negative control group that received either normal saline or phosphate buffered saline. The second group was treated with cisplatin alone at 4 mg/kg body weight, with each treatment cycle consisting of 4 days of once-daily intraperitoneal (i.p.) injection of cisplatin followed by 10 days of recovery. The third group was treated with cisplatin and NK cells in combination, with 4 mg/kg body weight of cisplatin, and each treatment cycle consisted of 4 days of once-daily i.p. injection of cisplatin followed by 10 days of recovery, along with NK cells being injected into tail vein on days 4 and 7 during the recovery period [once-daily intravenous (i.v.) injection of 1 × 10^7^ NK cells (> 98% viability)] [25]. Finally, the fourth group was treated with NK cells alone, with once-daily i.v. injections of 1 × 10^7^ NK cells on days 4 and 7 during the recovery period and no injections of cisplatin. Cisplatin was dissolved in 100 μl normal saline, and NK cells were mixed with 100 μl phosphate buffered saline. Two days after the last dose, we immediately collected blood samples from the mice after anesthesia with isoflurane. Then the mice were sacrificed by cervical dislocation. The tumor, thymus, liver, and spleen were all removed and weighed. The tumor volume (V) and body weight were measured every 3 days (V = 1/2 ab^2^, where “a” represents tumor length and “b” represents tumor width). For animal welfare and ethical reasons, micewere sacrificed after anesthesia when tumor size exceeded 2000 mm^3^. The tumor inhibitory rate was determined using the following formula: (1-a/b) 100%, where “a” represents the average tumor volume in the experimental group, while “b” represents the average tumor volume in the control group. The tumor growth ratio was calculated based on the formula: V_t_/V_0_, where “V_t_” represents the post-treatment tumor volume, while “V_0_” represents the pre-treatment tumor volume, which was measured on day 7. The organ weight/body weight ratio was used to calculate organ indices.

### Reagents and materials

BAX, β-actin, GAPDH, IL-2, IL-10, and CD4 antibodies were purchased from Proteintech (China). Caspase-3 antibody was purchased from Abcam (UK). Rae1 antibody was purchased from Santa Cruz (USA). Anti-rabbit or mouse antibody for western blotting analysis was obtained from KPL(USA). Cisplatin was obtained from MedChemExpress (New Jersey, USA).

### Cell culture

NK cells from healthy human umbilical cord blood were purchased from Hebei INcele Biotechnology Co. Ltd. (Shijiazhuang, China), identified by STR, tested for bacteria, fungi, mycoplasma and endotoxin, and NK cells phenotype CD3-CD16 + CD56 + detected by flow cytometry. NK cells were pre-activated by IL2 during culture. The mouse bladder cancer cell line MB49 was purchased from American Type Culture Collection (ATCC, Manassas, VA). MB49 cells was cultured in Dulbecco’s Modified Eagle’s (DMEM) media (Invitrogen, Grand Island, NY, USA) with 10% fetal bovine serum (GIBCO) in an incubator at 37 °C with 5% CO_2_.

### Histological analysis

Fresh renal tissues from mice were fixed in formalin and embedded in paraffin. Hematoxylin–eosin (H&E) staining was used to stain 4-μm-thick sections. Under blinded conditions, a pathologist evaluated the kidneys for clear signs of tubular injury, such as tubular dilatation, cytoplasmic vacuoles, exfoliation of epithelial cells, and cast formation. Image J software was used to analyze the images (NIH, Bethesda, MD, USA).

### Immunohistochemistry

Fresh spleen and tumor tissues from mice were embedded in paraffin after being fixed in formalin. Cut into 4 μm thick pieces were conducted following the methods for immunohistochemical analysis. These sections were dewaxed with xylene, rehydrated, and pre-incubated with 10% normal goat serum (KPL, USA) before being incubated overnight with primary antibodies at 4 °C. They were subsequently incubated with secondary antibody (horseradish peroxidase-labeled IgG antibody, KPL, USA). The color development process was performed using the DAB substrate kit. Sections were ultimately retained with hematoxylin. Images were captured with a microscope (Leica Microsystems, Mannheim, Germany), and Image J software was used to estimate the mean density of these cells (NIH, Bethesda, MD, USA).

### Western blotting analysis

RIPA buffer with protease inhibitors was used to lyse tissues and cells. 30 μg protein samples were loaded onto a 12% SDS/PAGE gel, then transferred to PVDF membranes (Millipore, Billerica, MA). These membranes were blocked in 5% skim milk for 2 h at room temperature before being incubated with appropriate dilutions of primary antibodies overnight at 4 °C. The membranes were then incubated for an additional 85 min at room temperature with secondary rabbit or mouse antibodies. In the end, the blots were visualized by reaction with ECL (chemiluminescence) Fuazon Fx (Vilber Lourmat).

### Quantitative real-time polymerase chain reaction (qRT-PCR)

Using the Trizol reagent (Invitrogen, Grand Island, NY), total RNA was extracted from tissues or cells as per the manufacturer’s instructions. RNA was used for reverse transcription using Superscript III transcriptase (Invitrogen). We determined the mRNA expression levels using a Bio-Rad CFX96 system with SYBR green. The qRT-PCR protocol was as follows: 95 °C for 5 min, followed by 40 cycles of 95 °C for 30 s, and 60 °C for 15 s. The 2^−ΔΔCt^ algorithm was applied to determine the relative expression levels of transcripts using GAPDH as a reference. Online Resource 1 lists the primer sequences in detail.

### ELISA

ELISA kits (Multisciences, Hangzhou, China) were used to evaluate the expression levels of IL-2, IFN-γ, IL-10, and TGF-β in serum and the lysates of spleen and tumor tissues in accordance with the manufacturer’s instructions.

### Flow cytometric analysis

The spleen’s immune cells were examined using flow cytometry. After washing twice with PBS, the spleen was cut into small pieces. The dissociated tissue was gently passed through a 70 μm nylon filter (Beyotime, Shanghai, China) to prepare a plesnocyte suspension. Cells resuspended in PBS were incubated with the following antibodies: CD45-Percp, CD3-FITC, CD4-Pecy7, CD11b-FITC, Gr-1-APC, and CD11c-PE (Elabscience Biotechnology Co., Ltd, Wuhan, China) in accordance with the manufacturer’s instructions. The stained cells were detected using a FACS CantoTM II instrument (BD Biosciences, San Diego, CA, USA). The data obtained were analyzed using FlowJo v10.8.1 software (Tree Star, San Carlos, CA, USA).

### Statistical analysis

Statistical analyses were performed using the GraphPad Prism 8 (GraphPad Software, San Diego, CA, USA). Data were presented as mean ± SD. All experiments were duplicated for three times. Student's t test, One-way ANOVA, or two-way ANOVA was used for statistical analysis. Survival curves were analyzed using the Kaplan–Meier method, and the significance of differences was determined using the log-rank test. All differences were deemed statistically significant at *p* < 0.05.

## Results

### Cisplatin combined with NK cells further inhibited murine bladder cancer tumor growth

**Fig. 1:**
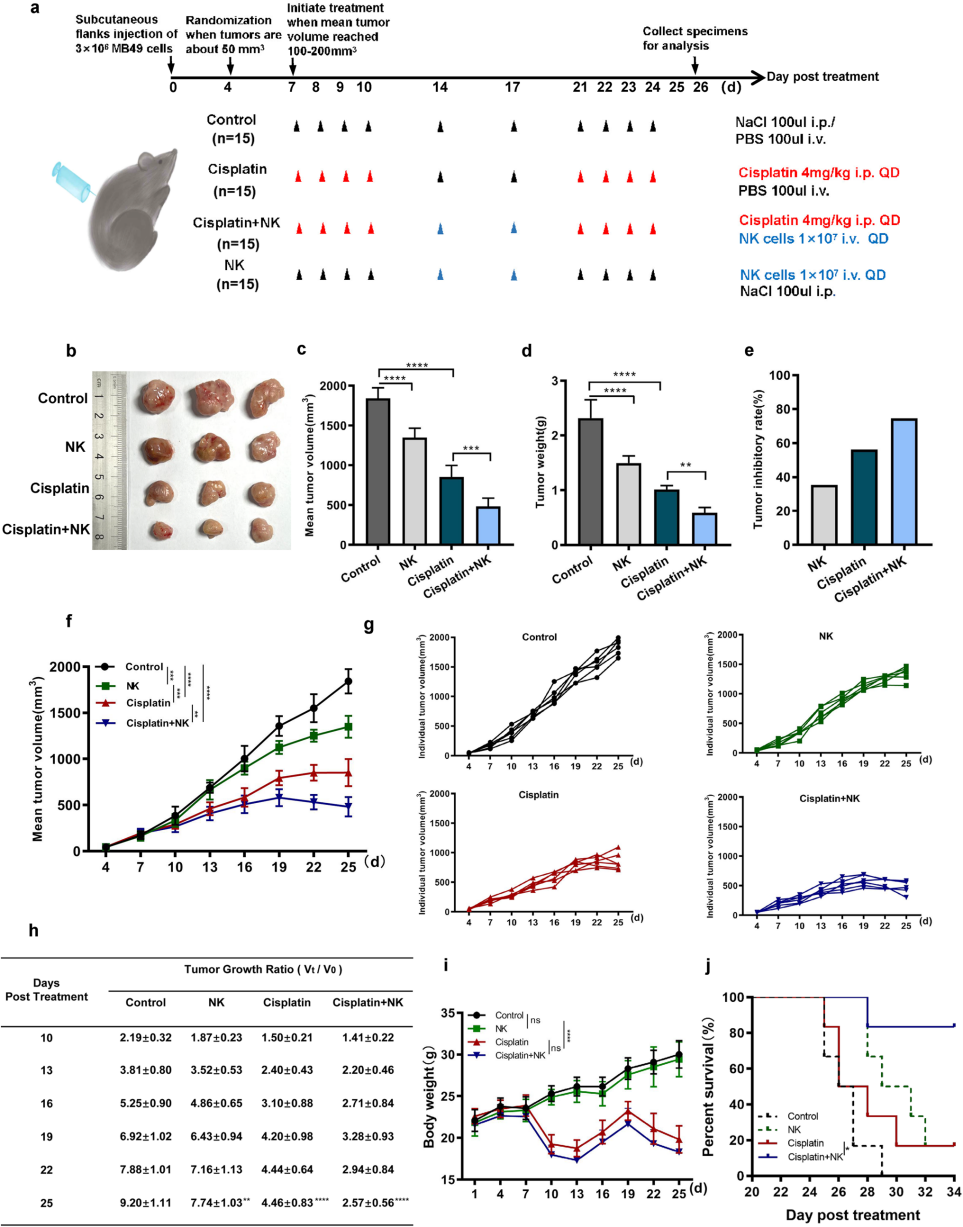
Cisplatin combined with NK cells further inhibited murine bladder cancer tumor growth. **a** Schematic representation of the experiment design investigating the effect of cisplatin and NK cells in combination on the treatment of MB49 tumor-bearing mice; **b** Representative images of transplanted subcutaneous tumors in all groups after 26 days of treatment; **c**–**e** Tumor volume, tumor weight, and tumor inhibitory rate in each group are displayed (n = 6); **f** Average MB49 tumor growth curves in C57BL/6N mice (n = 6) treated with PBS or NaCl control, NK cells, cisplatin, or cisplatin and NK cells in combination. Tumor growth was measured every 3 days; **g** Individual tumor volume growth curves; **h** Tumor growth ratio in C57BL/6N mice. **i** Body weight of each group measured every 3 days (n = 6); **j** Kaplan–Meier survival curves for mice (n = 6). Data are presented as mean ± SEM. ns = non-significant, *p < 0.05, **p < 0.01, ***p < 0.001, ****p < 0.0001 as determined by one-way ANOVA (**c**, **d**), two-way ANOVA (**f**, **h**, **i**), or log-rank test (**j**)

To examine the effects of cisplatin and NK cells on tumor growth, we created an MB49 bladder cancer transplantation model in C57BL/6N mice (Fig. 1a). Notably, after 26 days of treatment, mice treated with cisplatin or NK cells alone exhibited tumor growth suppression compared to untreated mice, whereas the group treated with cisplatin combined with NK cells exhibited dramatic tumor growth suppression (Fig. 1b). Tumor volume in the group treated with cisplatin combined with NK cells was obviously decreased compared with that in the other groups, while tumor weight data demonstrated similar antineoplastic efficacy as the tumor volume results (Fig. 1c, d). In particular, the combined treatment group achieved a tumor suppression rate of 74.6% (Fig. 1e). In addition, the tumor volume was monitored every 3 days during the treatment, with tumor growth curves analyzed at different times among the four groups and the tumor growth trend of each individual mouse in each group displayed. In the control group, the tumor volume showed a gradual increase over time. However, both the NK cells treatment group and the cisplatin treatment group exhibited slower tumor growth compared to the control group. The combined treatment group demonstrated the slowest tumor growth rate. It was found that the combination treatment enhanced tumor growth suppression (Fig. 1f, g). As depicted in Fig. 1h, we assessed the tumor growth ratio at various time points following administration of the treatment. The control group exhibited a significantly higher tumor growth ratio compared to the other three groups. The combined treatment group displayed the lowest tumor growth ratio. Interestingly, during the later stages of the experiment, the combined treatment group showed a gradual reduction in tumor growth ratio, leading to a decrease in tumor volume. The combination of NK cells and cisplatin had significantly controlled the speed of tumor growth. After 26 days of the experiment, the mice weighed approximately 29 g in the control group and NK cells treated group, but their body weights were significantly reduced in the cisplatin treated group (4 mg/kg) and the combination treated group (Fig. 1i). The results clearly showed that cisplatin was highly cytotoxic and that NK cells were unable to restore weight. Because of the high toxicity, mice treated with cisplatin alone had a significant mortality rate, whereas there was a notably higher survival rate in the combination group (Fig. 1j). In general, combined application of NK cells further suppressed tumor growth and improved the survival rate.

### NK cells protected against cisplatin-induced kidney injury and bone marrow suppression

**Fig. 2:**
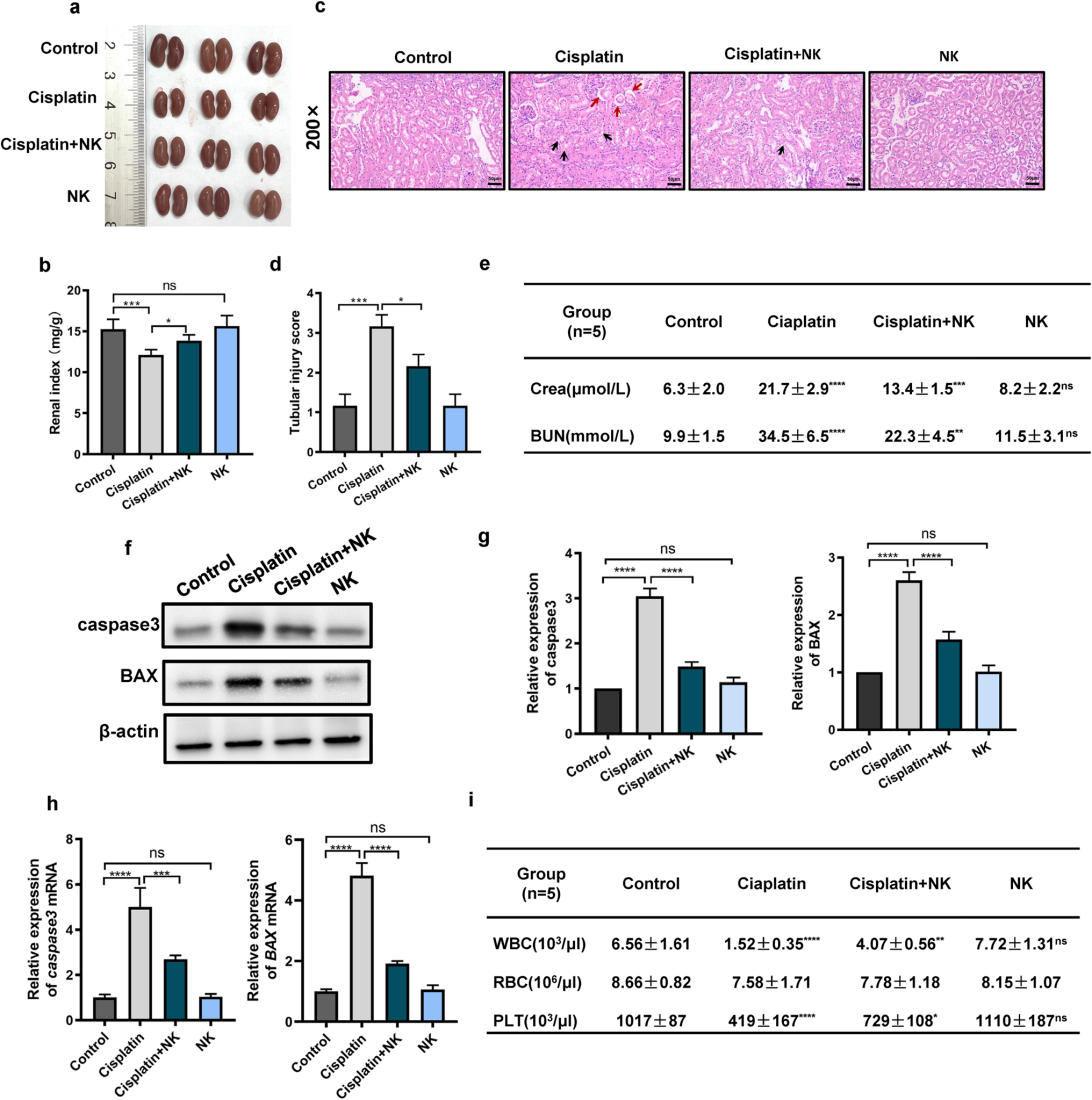
NK cells protected against cisplatin-induced kidney injury and bone marrow suppression. **a** Representative images of the kidney in all groups after 26 days of treatment; **b** Effects of cisplatin, NK cells, or cisplatin and NK cells in combination on the renal index in C57BL/6N mice (n = 6); **c** Photomicrographs of sections of kidney from different groups. The group given cisplatin shows: cytoplasmic vacuoles (black arrow) and exfoliation of epithelial cells (red arrow). Scale bar = 50 μm; **d** Tubular injury score in mice. **e** Serum creatinine and blood urea nitrogen levels were measured; **f**, **g** Western blotting of the expression levels of apoptosis-associated proteins in kidney tissue; **h** qRT-PCR of the expression levels of apoptosis-associated genes in kidney tissue; **i** Effect of cisplatin, NK cells, or cisplatin and NK cells in combination on hematopoietic function. Data are presented as mean ± SEM. Each experiment was repeated three times. *ns* non-significant, *p < 0.05, **p < 0.01, ***p < 0.001, ****p < 0.0001 as determined by one-way ANOVA (**b**, **d**, **e**, **g**, **h**, **i**)

Unfortunately, in a clinical context, cisplatin can have side effects on normal tissue. Kidney injury is a common complication in patients with cancer receiving cisplatin chemotherapy because cisplatin accumulates preferentially in the renal tubules [26, 27]. Renal insufficiency occurs in approximately 25%-40% of patients after cisplatin treatment [28]. To evaluate the effect of NK cells on renal injury induced by cisplatin, we dissected the kidney and determined the renal index (Fig. 2a, b). The renal index in cisplatin treated group was lower than that in the negative control group. However, cisplatin combined with NK cells treatment improved the renal index. We used histological analysis to assess the extent of tubular damage (Fig. 2c, d), such as tubular dilatation, cytoplasmic vacuoles, exfoliation of epithelial cells, and cast formation. Compared with the control group, the cisplatin treated group showed clear signs of tubular damage, including cytoplasmic vacuoles and exfoliation of epithelial cells. We did not find significant tubular dilatation and cast formation in the fields of view. Strikingly, the combined treatment group exhibited an improvement of tubular structure and a reduction in cytoplasmic vacuoles and exfoliation of epithelial cells. There were no significant differences between the control group and the NK cells treated group. Increased serum creatinine (Crea) and blood urea nitrogen (BUN) levels in the cisplatin treated group were dramatically reduced by combination treatment, which was consistent with the alteration in renal morphology (con vs. cisplatin: Crea, 6.3 ± 2.0 vs. 21.7 ± 2.9; BUN, 9.9 ± 1.5 vs. 34.5 ± 6.5. cisplatin vs. cisplatin + NK: Crea, 21.7 ± 2.9 vs. 13.4 ± 1.5; BUN, 34.5 ± 6.5 vs. 22.3 ± 4.5) (Fig. 2e). Similarly, cisplatin also caused liver damage, and the administration of NK cells improved liver function (Online Resource 2). To further evaluate the protective effect of NK cells on the kidney, the expression of the apoptosis-related proteins BAX and caspase-3 was measured via western blotting. The results showed that the protein expression increased in the cisplatin treated group compared with that in the control group, while in the combination treated group there was a clear downregulation of the protein expression compared with that in the cisplatin treated group (Fig. 2f, g). Additionally, once NK cells were coupled with chemotherapy, the increased mRNA expressions of BAX and caspase-3 caused by cisplatin were noticeably reduced (Fig. 2h). Myelosuppression is another common side effect of cisplatin. To evaluate this, peripheral blood cells were counted in the mouse models. Leukocytes and platelets were notably higher in the combination treated group than in the cisplatin treated group, while no significant difference in erythrocytes was noted (con vs. cisplatin: WBC, 6.56 ± 1.61 vs. 1.52 ± 0.35; PLT, 1017 ± 87 vs. 419 ± 167. cisplatin vs. cisplatin + NK: WBC, 1.52 ± 0.35 vs. 4.07 ± 0.56; PLT, 419 ± 167 vs. 729 ± 108) (Fig. 2i). These findings demonstrated that NK cells could alleviate the organ damage caused by cisplatin in vivo.

### NK cells relieved immune organ damage and improved immune cell responses

**Fig. 3:**
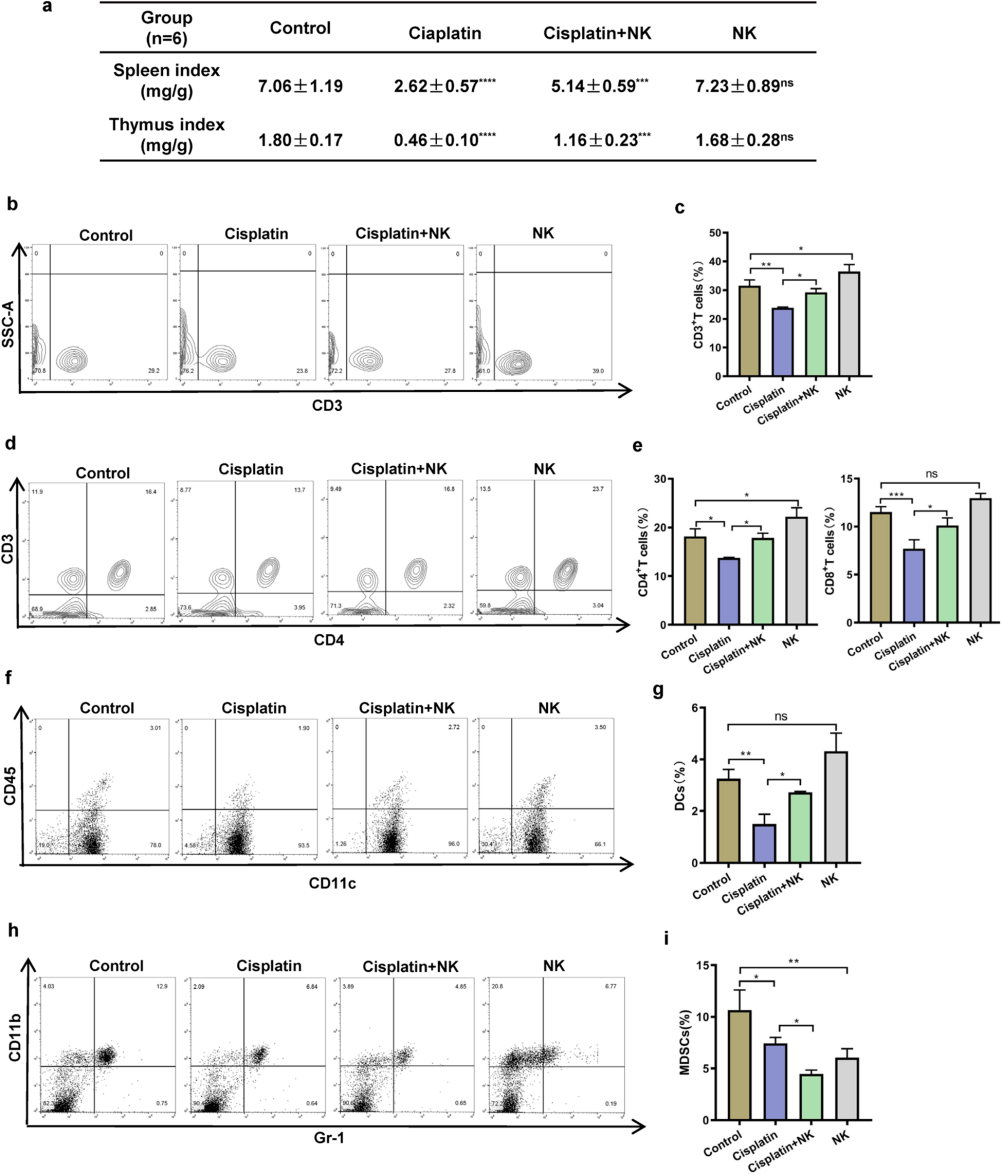
NK cells relieved immune organ damage and improved immune cell responses. **a** Effect of cisplatin, NK cells, or cisplatin and NK cells in combination on the spleen index and thymus index in C57BL/6N mice (n = 6). Immunomodulatory effects of NK cells on T cells (**b**, **c**, **d**, **e**), CD45 + CD11c + DCs (**f**, **g**), and CD11b + Gr-1 + MDSCs (**h**, **i**) in spleen immunocytes. Data are presented as mean ± SEM. Each experiment was repeated three times. *ns* non-significant, *p < 0.05, **p < 0.01, ***p < 0.001, ****p < 0.0001 as determined by one-way ANOVA (**a**, **c**, **e**, **g**, **i**)

One of the main side effects of chemotherapy medications appears to be immunosuppression. The spleen and thymus are important immune organs [29]. Therefore, to assess the effects of cisplatin and NK cells on immune functions, we determined the immune organ indices of C57BL/6N mice (Fig. 3a). The thymus and spleen indices were clearly lower in the cisplatin treated group than in the control group. When NK cells were combined with cisplatin treatment, these indices improved compared to cisplatin treatment alone, indicating that, to some extent, NK cells reversed the immunosuppressive effects of cisplatin on immune organs. After the administration of cisplatin treatment, the proportion of CD3 + T lymphocytes drastically decreased when compared with that in the control group. The combination treatment group had a higher percentage of CD3 + T lymphocytes than the cisplatin treated group (Fig. 3b, c). Additionally, the proportions of CD3 + CD4 + T lymphocytes and CD3 + CD8 + T lymphocytes decreased in the cisplatin treated group. In contrast to those in the group treated with cisplatin alone, the proportions of CD3 + CD4 + T lymphocytes and CD3 + CD8 + T lymphocytes increased with NK cells supplementation (Fig. 3d, e). Similarly, after cisplatin treatment, the proportions of CD45 + CD11c + dendritic cells (DCs) and CD11b + Gr-1 + myeloid-derived suppressor cells (MDSCs) cells drastically decreased compared with those in the control group. Moreover, the proportion of DCs was higher in the group treated with cisplatin combined with NK cells than in the group treated with cisplatin. However, the proportion of MDSCs was lower in the combined treatment group than in the cisplatin treated group (Fig. 3f–i). All of these results demonstrated that administering NK cells induces immune cell responses.

### NK cells promoted cytokine production

**Fig. 4:**
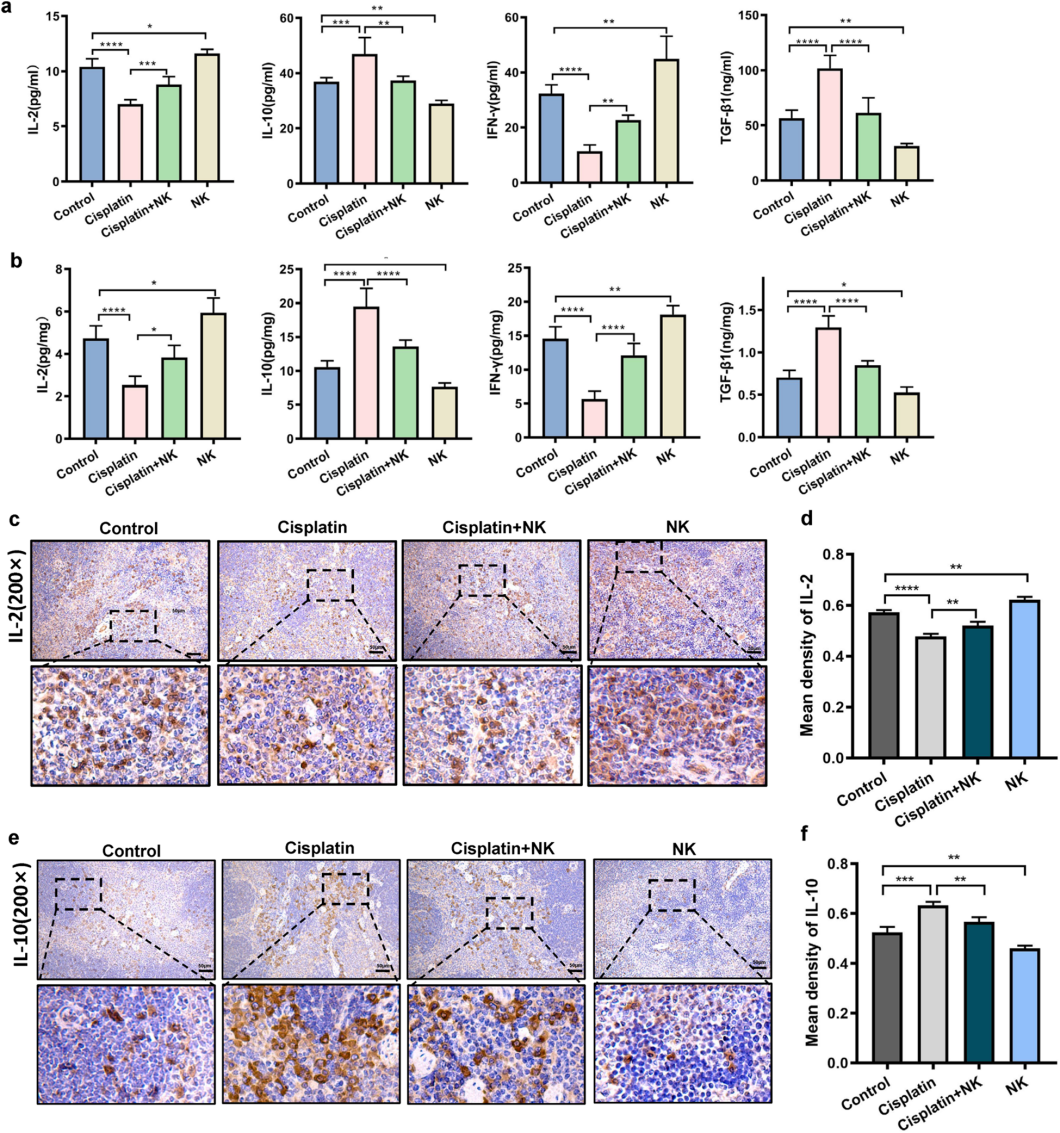
NK cells promoted cytokine production. Effects of cisplatin, NK cells, or cisplatin and NK cells in combination on IL-2, IFN-γ, IL-10, and TGF-β expression in serum (**a**) and spleen tissue (**b**). Representative images of IL-2 (**c**, **d**) and IL-10 (**e**, **f**) staining of spleen tissue in all groups. Scale bar = 50 μm. Data are presented as mean ± SEM. Each experiment was repeated three times. *ns* non-significant, *p < 0.05, **p < 0.01, ***p < 0.001, ****p < 0.0001 as determined by one-way ANOVA (**a**, **b**, **d**, **f**)

To determine whether NK cells can improve the immunological function of MB49 tumor-bearing mice, the levels of cytokines in their serum and spleen tissues were determined using ELISA. Compared to the control group, cisplatin therapy considerably reduced the expressions of IL-2 and IFN-γ, whereas NK cell treatment significantly increased them. Serum IL-2 and IFN-γ levels were higher in the group treated with cisplatin combined with NK cells than in the group treated with cisplatin alone (Fig. 4a). The outcomes additionally demonstrated that changes in IL-2 and IFN-γ expression in spleen tissues matched those in serum (Fig. 4b). Regardless of whether serum or spleen tissue was analyzed, the IL-10 and TGF-β levels were higher in the cisplatin treated group than in the control group, whereas they were lower in the NK cells treated group. Meanwhile, compared the cisplatin treated group, the IL-10 and TGF-β levels were notably downregulated in the combination treatment group (Fig. 4a, b). Finally, mouse spleen specimens were assessed immunohistochemically for IL-2 and IL-10 levels (Fig. 4c–f). As predicted, IL-2 was abundant in the NK cells treated group, while its expression level was lowest in the cisplatin treated group. The expression level of IL-2 was higher in the group treated with cisplatin combined with NK cells than in the group treated with cisplatin alone. On the contrary, the changes of IL-10 expression were opposite to those of IL-2. Overall, these findings showed that NK cell therapy had a positive impact on the immune function of MB49 tumor-bearing mice.

### NK cells enhance the immune response in tumor tissue from mice

**Fig. 5:**
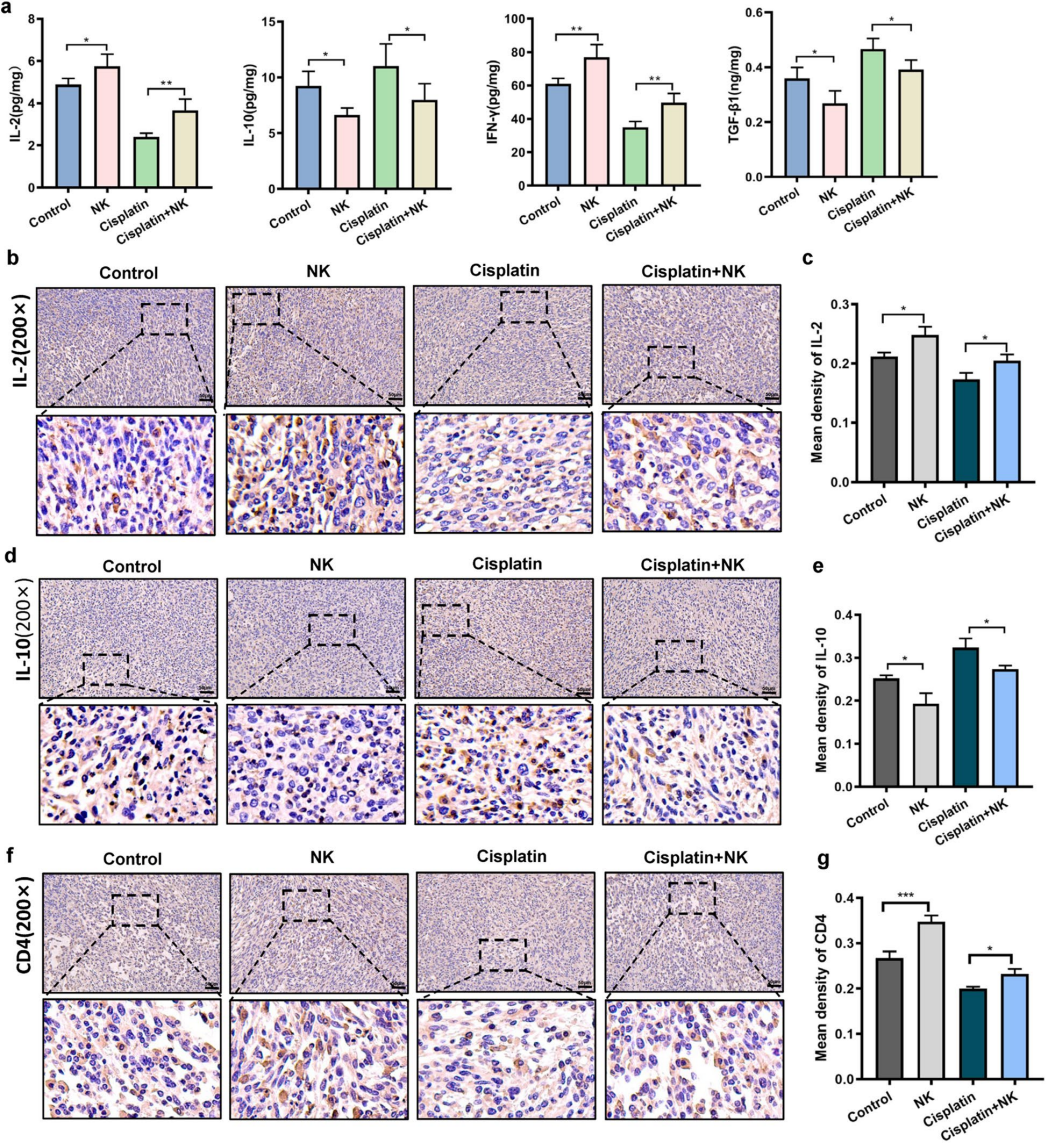
NK cells enhance the immune response in tumor tissue from mice. **a** Effects of cisplatin, NK cells, or cisplatin and NK cells in combination on IL-2, IFN-γ, IL-10, and TGF-β expression in tumor tissue. Representative images of IL-2 (**b**, **c**), IL-10 (**d**, **e**) and CD4 (**f**, **g**) staining of tumor tissue in all groups. Scale bar = 50 μm. Data are presented as mean ± SEM. Each experiment was repeated three times. *ns* non-significant, *p < 0.05, **p < 0.01, ***p < 0.001, ****p < 0.0001 as determined by one-way ANOVA (**a**, **c**, **e**, **g**)

A key line of defense against tumors is the immune system, and immune cells have a close relationship with antitumor immunity [30]. Cytokines also play a role in antitumor defense. In this investigation, we further assessed how NK cells affect the immune response in tumor tissues. First, we used the ELISA to detect cytokines in tumor tissues (Fig. 5a). The expressions of IFN-γ and IL-2 were clearly higher in the NK cells treated group than in the negative control group. Moreover, the expressions of IFN-γ and IL-2 in the cisplatin treated group were restored by NK cells combined treatment. Conversely, the expressions of IL-10 and TGF-β in the NK cells treated group dramatically decreased when compared to that in the control group. The group treated with cisplatin combined with NK cells had lower levels of IL-10 and TGF-β than the group treated with cisplatin alone. Second, mouse tumor specimens were assessed for IL-2, IL-10, and CD4 levels by immunohistochemistry. The expression changes of IL-2 and IL-10 were consistent with those revealed by ELISA (Fig. 5b–e). Besides, there was an abundance of CD4 in the NK cells treated group. Cisplatin combined with NK cells led to higher CD4 protein expression level than cisplatin alone (Fig. 5f, g). NK cells enhanced the infiltration of immune cells and cytokines in tumor tissues and thus improved the immune response.

### Cisplatin increased the expression of NKG2D ligands

**Fig. 6:**
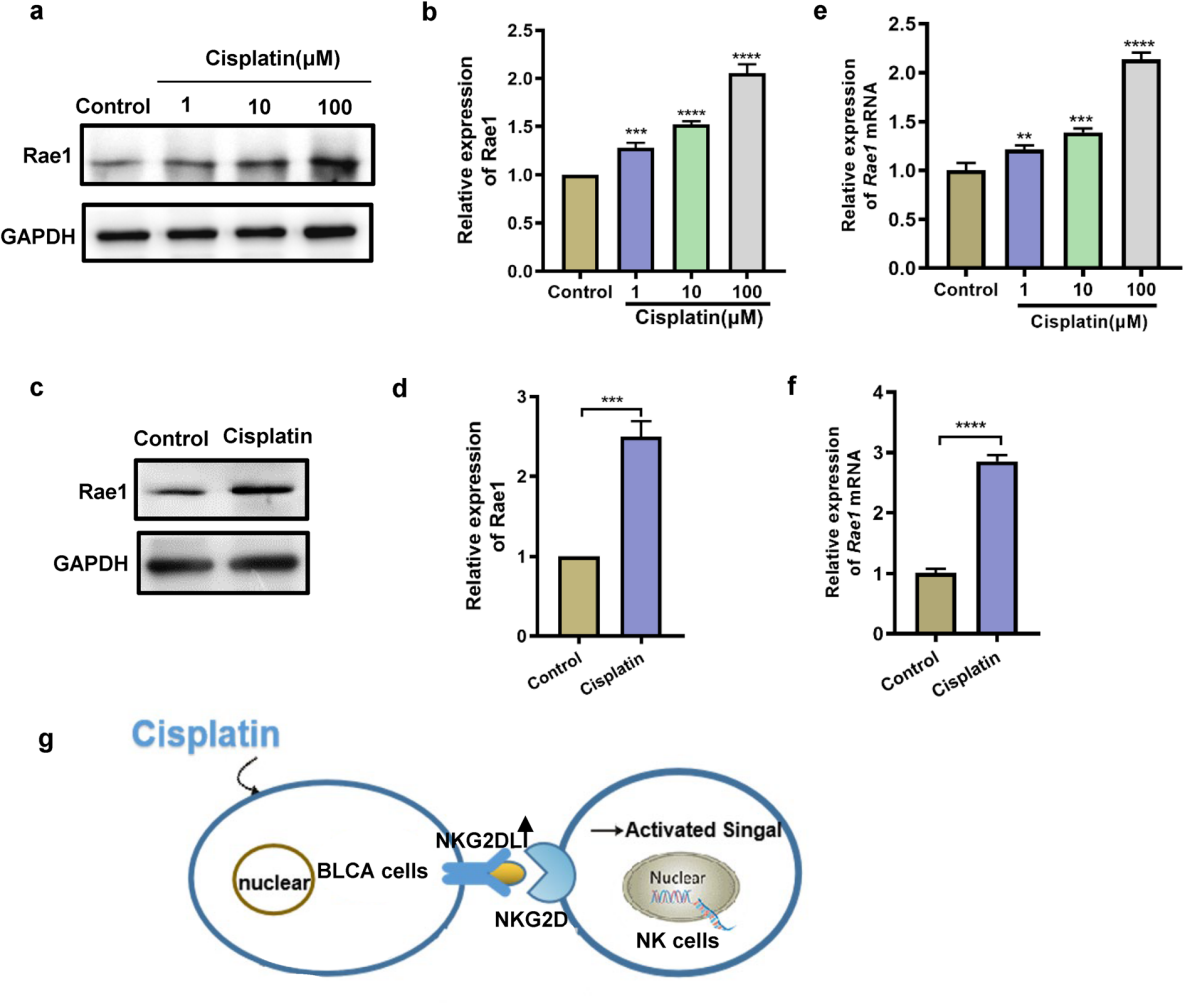
Cisplatin increased the expression of NKG2D ligands. **a**, **b** Western blotting was used to detect Rae1 in lysed MB49 cells exposed to cisplatin for 24 h. **c**, **d** Western blotting was used to detect Rae1 in tumor tissues. **e**, **f** qRT-PCR of the expression levels of Rae1 in cells and tumor tissues. **g** Schematic representation of the pathway by which cisplatin regulates bladder cancer cells. Data are presented as mean ± SEM. Each experiment was repeated three times. *ns* non-significant, *p < 0.05, **p < 0.01, ***p < 0.001, ****p < 0.0001 as determined by one-way ANOVA (**b**, **e**) and unpaired t-test (**d**, **f**)

The expression of the activating receptor, natural killer group 2, member D (NKG2D), on the surface of NK cells influences the effectiveness of the antitumor immune response [31]. MHC class I chain related A (MICA) is a ligand for NKG2D, which is expressed on many human tumor cells. Similarly, retinoic acid early inducible 1 (Rae1) is a murine NKG2D ligand. The NKG2D ligands play a decisive role in antitumor immune surveillance. A previous report described that gemcitabine increased the expression level of MICA on HepG-2 cells and further enhanced NKG2D-mediated NK cells antitumor immunity [32]. Therefore, we hypothesized that cisplatin may upregulate the expression of NKG2D ligands on bladder cells. The results demonstrated a dose-dependent increase in Rae1 expression on MB49 cells (Fig. 6a, b). The expression of Rae1 in tumor tissues was also upregulated when treated with cisplatin (Fig. 6c, d). Furthermore, the mRNA expression changes of Rae1 in tumor tissues and MB49 cells were consistent with those revealed by western blotting (Fig. 6e, f). In particular, we discovered that cisplatin increased the expression of NKG2D ligands, which can stimulate the antitumor activity of NK cells (Fig. 6g).

## Discussion

Bladder cancer is one of the malignant tumors that seriously threaten people's health. The treatment of bladder cancer is mainly based on surgery. Chemotherapy, radiotherapy and biological therapy play an auxiliary role. A growing body of research supports the notion that chemotherapy and immunotherapy can be used in tandem to treat cancer by modulating the activity of various cytokines or immunocytes. Therefore, chemoimmunotherapy is considered a promising therapeutic approach aimed at promoting or stimulating immune responses as well as further inhibiting tumor growth.

Animal models of bladder cancer are an important tool for studying this disease, and they facilitate the clinical screening of anti-bladder tumor drugs. Here, we used an MB49 tumor-bearing mouse model to investigate the association between cisplatin and NK cells. In our experiment, NK cells were obtained from human umbilical cord blood, which exhibited low immunogenicity and excellent tissue compatibility. NK cells were not HLA-restricted, and their transplantation did not cause either acute or chronic graft-versus-host disease (GVHD) [33, 34]. To select cisplatin dose, once daily (3 mg/kg or 3.5 mg/kg) for 4 days followed by 10 days of recovery was used in some studies [35, 36]. Through the preliminary experiment, the dosage of cisplatin chosen in the experiment was once daily (4 mg/kg) for 4 days followed by 10 days of recovery. The mice receiving cisplatin chemotherapy began to die successively after the second cycle of administration. Thus two days after the last dose, specimens were collected from the mice after anesthesia for further analyses. First, we found that treatment with NK cells or cisplatin alone could inhibit tumor growth, but cisplatin had superior antitumor activity compared to the NK cells treatment group. NK cells combined with cisplatin had better antitumor activity. A tumor suppression rate of 74.6% was achieved in the group treated with this combination. This was related to the superimposed effect of cisplatin and NK cells, and there might be a certain synergistic effect between them. The cisplatin toxicity made the mice unable to withstand successive cycles of chemotherapy and the survival rate of the cisplatin treated group was decreased. Shorter survival was remarkably improved in the combination treatment group. In terms of tumor volume, cisplatin had a higher tumor inhibition rate than the control group, while the survival rate was not significantly improved. It could be due to organism damage caused by cisplatin toxicity.

Immunosuppression and organ damage are the two main toxicities associated with cisplatin. The most major side effect of cisplatin is nephrotoxicity since it is primarily excreted from the kidney. By causing oxidative stress, inflammation, mitochondrial malfunction, and apoptosis, cisplatin damages the kidneys [37]. We assessed the extent of kidney injury by measuring the expressions of the apoptosis-related proteins caspase-3 and BAX. The expressions of caspase-3 and BAX were markedly reduced after the combined treatment. Moreover, NK cells combined with cisplatin could restore the Crea and BUN levels. These findings suggest that NK cells exert a protective effect on renal function. Cisplatin can also damage the liver, heart, intestine, and other organs. We have described the protective effects of NK cells on the liver in the Supplemental Material, but the effects on other organs need to be confirmed. Notably, myelosuppression was also remarkably alleviated in the combination treated group.

In this research, NK cells clearly increased the spleen and thymus indexes and enhanced the immune response, which would reduce the immune cytotoxicity caused by cisplatin. Following NK cells therapy, higher proportions of CD3 + CD4 + T lymphocytes, CD3 + CD8 + T lymphocytes, and DCs were confirmed in the splenocytes of MB49 tumor-bearing mice. Meanwhile, NK cells reduced the proportion of MDSCs. We found that TGF-β and IL-10 were significantly downregulated in the tumor microenvironment with the NK cells treatment. The increased expression of MDSCs in the tumor microenvironment suggests that they participate in tumor immune escape through immunosuppression. Previous research shows that TGF-β and IL-10 can suppress T lymphocytes activity and proliferation, resulting in the failure of immunological responses against tumor [38]. Therefore, these results reasonably explained the application of NK cells to enhance antitumor activity. IL-2 has been reported to improve immunity and promote the destruction of autologous tumors [39], while IFN-γ has potent anticancer and antiangiogenic properties. Moreover, by controlling the expression of the c-myc gene, IFN-γ can slow the proliferation of cancer cells [40]. We also found that NK cells significantly promoted the levels of the proinflammatory cytokines IL-2 and IFN-γ. IL-2 and IFN-γ were upregulated in the tumor microenvironment in the combination treated group, as were CD4 + T lymphocytes. It can be hypothesized that NK cells enhanced immune cell responses and promoted cytokine production in the MB49 tumor-bearing mouse model.

NKG2D is a type II transmembrane protein with an extracellular domain that resembles a C-type lectin, which is an activating receptor present on the surface of NK cells and T cells [41, 42]. After NKG2D binds to ligands, the PI3K and Grb2-vavl complexes are activated, which activates downstream Akt kinases [43], enhancing the cytotoxicity of NK cells. The antitumor activity of NK cells is critically dependent on the relationship between NKG2D and ligands. A previous report described that gemcitabine increased the expression level of MICA on HepG-2 cells and further enhanced NKG2D-mediated NK cells antitumor immunity. Our research revealed that cisplatin elevated the expression of NKG2D ligands on bladder cancer cells. This then stimulated NKG2D and activated the antitumor capacity of NK cells. This provides novel and strong evidence for the value of using NK cells and cisplatin in combination.

A prospective cohort study evaluated the efficacy and safety of NK cells therapy in combination with chemotherapy in patients with locally advanced colon carcinoma [44]. They found that NK cells infusion combined with chemotherapy was well tolerated and showed great potential for the prevention of recurrence and prolonging of survival. NK cells therapy provided a novel and practical strategy for the treatment of patients with locally advanced colon carcinoma. The polysaccharide SEP had been reported to enhanced antitumor activity of gemcitabine against liver cancer through activating NK cells. Meanwhile, gemcitabine upregulated MICA expression and attenuated soluble MICA secretion, which in turn enhanced the cytotoxicity of NK cells against cancer cells [32]. Besides, some studies reported that NK cells in combination with inhibitors enhanced the control and treatment of tumors [45, 46]. These data indicated that combining NK cells therapy represented a compelling approach in the treatment of cancer, offering great potential for therapeutic success. Chemoimmunotherapy is a promising strategy to improve the therapeutic efficacy, which could solve the problem of clinical failure of conventional chemotherapy to some extent. It is widely acknowledged that cisplatin significantly inhibits bladder cancer growth. Our study confirmed that NK cells combined with cisplatin is significantly better than cisplatin monotherapy for treating bladder cancer. Our data offer the first proof of the immunotherapeutic potential of NK cells in combination with cisplatin.

## Conclusions

In conclusion, NK cells can restore immune organs, regulate cellular immune responses, and increase cytokine levels in order to achieve anticancer activity. The synergy between NK cells and cisplatin can further suppress the growth of bladder tumors and alleviate the side effects of cisplatin treatment in tumor-bearing mice, providing an appropriate chemotherapeutic immune adjuvant.

## Supplementary Information

Below is the link to the electronic supplementary material.

Supplementary file1 (PDF 70 KB)

Supplementary file2 (PDF 383 KB)

## Data Availability

The data used to support the findings of this study are included within the article and its supplementary materials.
