# Supplementary material for: Natural killer cells strengthen antitumor activity of cisplatin by immunomodulation and ameliorate cisplatin-induced side effects
Source: Int Urol Nephrol. 2023 May 30;55(8):1957–70. doi: 10.1007/s11255-023-03650-w (PMC10329593; doi:10.1007/s11255-023-03650-w)
Supplement: Supplementary file 1 — Supplementary file1 (PDF 70 KB) [file 11255_2023_3650_MOESM1_ESM.pdf]

Natural killer cells strengthen antitumor activity of cisplatin by immunomodulation and ameliorate cisplatin-induced side effects

Zhu Wang, Zhan Yang, Changbao Qu, Jinmin Li, and Xiaolu Wang

Correspondence: Xiaolu Wang, Department of Urology, The Second Hospital of Hebei Medical University, Shijiazhuang, China. E-mail: xiaoluwang311@163.com

Journal name: International Urology and Nephrology

Sequences of the primers for qRT-PCR

Gene	Primer Sequence (5′-3′)
Mouse caspase3	F: ATGGAGAACAACAAAACCTCAGT<br>R: TTGCTCCCATGTATGGTCTTTAC
Mouse BAX	F: TGGAGATGAACTGGACAGCAATAT<br>R: GCAAAGTAGAAGAGGGCAACCAC
Mouse Rae1	F: GCTGTTGCCACAGTCACATC<br>R: CCTGGGTCACCTGAAGTCAT
Mouse GAPDH	F: GTCTTCACTACCATGGAGAAGG<br>R: TCATGGATGACCTTGGCCAG
