# Supplementary material for: Natural killer cells strengthen antitumor activity of cisplatin by immunomodulation and ameliorate cisplatin-induced side effects
Source: Int Urol Nephrol. 2023 May 30;55(8):1957–70. doi: 10.1007/s11255-023-03650-w (PMC10329593; doi:10.1007/s11255-023-03650-w)
Supplement: Supplementary file 2 — Supplementary file2 (PDF 383 KB) [file 11255_2023_3650_MOESM2_ESM.pdf]

Natural killer cells strengthen antitumor activity of cisplatin by immunomodulation and ameliorate cisplatin-induced side effects

Zhu Wang, Zhan Yang, Changbao Qu, Jinmin Li, and Xiaolu Wang

Correspondence: Xiaolu Wang, Department of Urology, The Second Hospital of Hebei Medical University, Shijiazhuang, China. E-mail: xiaoluwang311@163.com

Journal name: International Urology and Nephrology

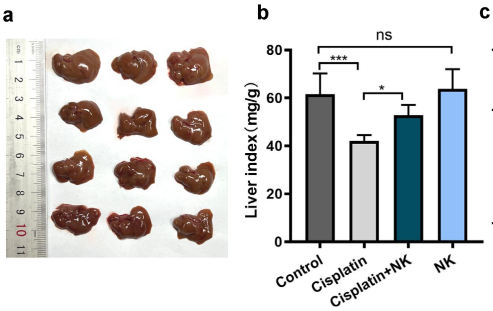

c

Group (n=5)	Control	Ciaplatin	Cisplatin+NK	NK
ALT(U/L)	48.0±10.3	176.8±51.5****	118.7±17.0*	62.2±11.3^{ns}
AST(U/L)	115.3±43.0	403.4±75.9****	275.0±50.5**	141.6±40.3^{ns}

NK cells protected against cisplatin-induced liver injury. (a) Representative images of liver in all groups after 26 days of treatment. (b) Effects of cisplatin, NK cells, or cisplatin and NK cells in combination on the liver index in C57BL/6N mice (n = 6). (c) Serum ALT and AST levels were measured. Data are presented as mean ± SEM. ns = non-significant, *p < 0.05, **p < 0.01, ***p < 0.001, ****p < 0.0001 as determined by one-way ANOVA (b, c).
